# The development of a tool for GPs to manage overweight and obesity in children: A Delphi study

**DOI:** 10.1080/13814788.2024.2413877

**Published:** 2024-10-21

**Authors:** Maxime Adriana Maria van der Velden, Madelon van Tilborg-den Boeft, Sylvia Buis, Wilma Jansen, Patrick Jan Eugène Bindels, Marienke van Middelkoop

**Affiliations:** a Department of General Practice, Erasmus MC Medical University Center Rotterdam, Rotterdam, The Netherlands; b Department of Public Health, Erasmus MC Medical University Center Rotterdam, Rotterdam, The Netherlands; c Department of Social Development, City of Rotterdam, Rotterdam, The Netherlands

**Keywords:** Children, overweight, obesity, general practice, tools, Delphi study

## Abstract

**Background:**

General practitioners (GPs) could play an important role in the management of overweight and obesity in children. However, GPs experience different barriers and are in need of supportive tools. In order to support GPs to identify, address and refer these children, new tools should be developed.

**Objective:**

To establish consensus among GPs about the content topics of a supportive tool to identify, address and refer children with overweight and obesity in general practice.

**Methods:**

A two-round Delphi study was conducted with GPs identified as experts. A concept of a supportive tool was constructed based on focus-group interviews with GPs, practice nurses and parents of children with and without overweight and literature. The tool was categorised into five topics: identifying, initiating and continuing weight-related conversations, referring and evaluating a tool manual. GPs evaluated statements on the tool’s topics in terms of importance. All statements were rated on a 5-point Likert scale and consensus was set at ≥70% of respondents agreeing with the statements.

**Results:**

GPs agreed that a supportive tool must contain a child’s specialised BMI calculator; examples to initiate and to continue weight-related conversations with parents and children; a map with available interventions; and a manual including information and resources about health risks of overweight and obesity during childhood.

**Conclusion:**

The content topics of a supportive tool for GPs to identify, discuss and refer children with overweight and obesity were determined through a consensus-driven process. Further validation and assessment are required through a feasibility and implementation study.

## KEY MESSAGES

General practitioners (GPs) are in need of support to manage overweight and obesity in children.

This paper establishes the content topics of a supportive tool for GPs to identify, address and refer children with overweight and obesity.

The feasibility of the supportive tool in daily general practice must be evaluated.

## Introduction

Overweight and obesity among children is an increasing health problem worldwide [1]. In the Netherlands, the number of children with overweight and obesity reached a percentage of 11.3% and 3.7% in 2023 [2]. Most children develop comorbidities associated with overweight or obesity (e.g. hypertension, dyslipidaemia) before receiving a formal diagnosis which have negative impacts on their health [3]. Furthermore, excessive weight during childhood often remains in adulthood which is associated with an increased risk to develop diseases like osteoarthritis, cardiovascular- and metabolic diseases [1]. Therefore, it is of great importance to address and manage overweight and obesity in children as early as possible.

In the Netherlands, general practitioners (GPs) are the first point of care, with most children aged four to 12 visiting their GP annually [4]. This makes general practice a unique setting for identifying and addressing overweight and obesity in children. Additionally, the Dutch Obesity guidelines for GPs state that GPs should discuss obesity in children during a consultation, regardless the consultation’s primary reason [5,6]. Also, parents consider general practice as well placed to address overweight in children, due to the longitudinal doctor-patient relationship and their confidence in the GPs medical expertise [7,8]. Parents value assessments of their child’s weight and appreciate weight-related conversations, provided that these are conducted in a non-judgemental and supportive manner [9].

GPs acknowledge that these tasks are within their professional responsibilities [10]. However, adherence to existing guidelines among GPs is low which may be attributed to the challenges GPs face like time constraints and difficulties in initiating weight-related conversations [1,11,12]. GPs seem to be afraid to compromise their doctor-patient relationships [5,7]. Moreover, GPs are often unaware of available referral options which leads to a notably low referral rate of children with overweight in general practice [13]. Equipping GPs with necessary tools to identify, address and refer children with overweight and obesity within a general practice is essential [10,14]. Therefore, this Delphi study aimed to establish consensus on the content topics to develop a supportive tool for GPs to identify, address and refer children with overweight and obesity in practice.

## Method

### Study design

An online, iterative Delphi study was conducted to establish consensus on the content topics of a supportive tool for GPs to identify, address and refer children with overweight and obesity. The Delphi procedure was chosen as it is a well-established technique to reach consensus on a topic among a group of experts. This procedure is characterised by two or more survey rounds with controlled feedback, group response and anonymity. A response rate of 70% is often used in Delphi studies to maintain the accuracy of this technique [15].

An initial concept of a supportive tool was developed based on (1) focus-group interviews with GPs, practice nurses and parents of children with and without overweight and obesity [10]; (2) literature on weight management strategies for children and adults in general practice [5,6,16–18].

### Panel of GP experts

To assure the supportive tool would address the needs and demands of Dutch GPs, it was decided that the expert panel should exist of Dutch GPs working in general practice for at least two days a week. Members of the Academic Network of General Practitioners of the Erasmus MC University Medical Centre and GP trainers of the vocational GP training were approached via email. They received information about the study, the need for engagement in both rounds and required time to complete each round. For a Delphi study, literature recommends a minimal group size of 30 experts [15].

### Data collection and analysis

An online survey was constructed using the software program Lime Survey. Two GPs from the department General Practice of Erasmus MC tested this survey and provided feedback regarding clarity of instructions, ease and time needed to complete the survey. Completing the first survey requires approximately 15 min and would be less for the second survey. No incentives were provided. All participating experts received an email with the link to the survey. The study was conducted between April and August 2023.

The surveys consisted of statements about the content of the tool, which were categorised into five topics: (1) identifying overweight; (2) initiating- and; (3) continuing weight-related conversations; (4) referring children; (5) evaluating the manual. Details on the statements within each topic are listed in Table 1. The first survey consisted of 32 statements and experts were asked to rate these statements on importance. A 5-point Likert scale was used, ranging from 1 = totally disagree to 5 = totally agree. At each statement an open-ended question was provided where experts were encouraged to provide suggestions or arguments that substantiated their opinion. For round two, each expert received next to the second survey an overview of the outcomes from the initial survey. This allowed them to review the results from the previous round, gaining insights into how opinions were distributed among experts and to reconsider and potentially adjust their opinion. Statements that did not reach consensus in round one were refined and suggested changes were adopted in the revised tool and included in the second survey after they were discussed among the research group (MvdV;MvM;MvT). Experts were asked to (re)score the statements on importance using the same 5-point Likert scale and open-ended questions were provided for suggestions. Both, the surveys and drafts of the tool are shown in Appendix 1, supplementary material.

**Table 1.** Overview of the main- and sub-topics of the statements in the two rounds of this Delphi study.

Main topics	Subtopics first round	Subtopics second round
1) Identifying	Flowchart to identify overweight and obesity	X
	BMI calculator	X
	BMI categories	X
	Waist circumference measurement	Waist circumference measurement
2) Initiating	Asking permission to parents	Asking permission to parents and children
	Involving the child	X
	Attitude	Attitude
	Choice of words	Choice of words
	Example sentences to initiate weight-related conversation	X
3) Continuing	Motivational interviewing	X
	Informing about health risks	X
	Resistance among parents	X
	Example sentences to continue weight-related conversation	Example sentences to continue weight-related conversation with parents and children
4) Referring	Intervention map	Intervention map
	Referral options (e.g. paediatrician, dietician, combined lifestyle intervention)	X
5) Evaluating a tool manual	Background information (e.g. diet, physical activity, sleep, screen time)	Background information (e.g. diet, physical activity, sleep, screen time; cultures and ethnicity; health risks of overweight)
	Information resources (e.g. poster, flyers, website links, papers, online)	Information resources (e.g. poster, flyers, website links, papers, online)

X = Consensus was achieved for the statements within the topic in the first round.

For both rounds the experts had one-month turnover time to complete the survey. Email reminders were sent at 2 weeks and 1 week before closing. In both rounds, consensus was reached when ≥70% of the experts scored a statement with ‘agree’ or ‘totally agree’. If no consensus was reached the research team made decisions on the remaining statements.

## Results

### Delphi study design and participants

A total of 33 GPs consented to participate and 31 of them completed the first survey (93.9% response rate). In round one, consensus was reached for 21 of the 32 statements. The second survey consisted of 10 modified statements and two additional statements. Round two was completed by 29 experts (93.5% response rate).

### Statements about identifying

**Figure 1.**
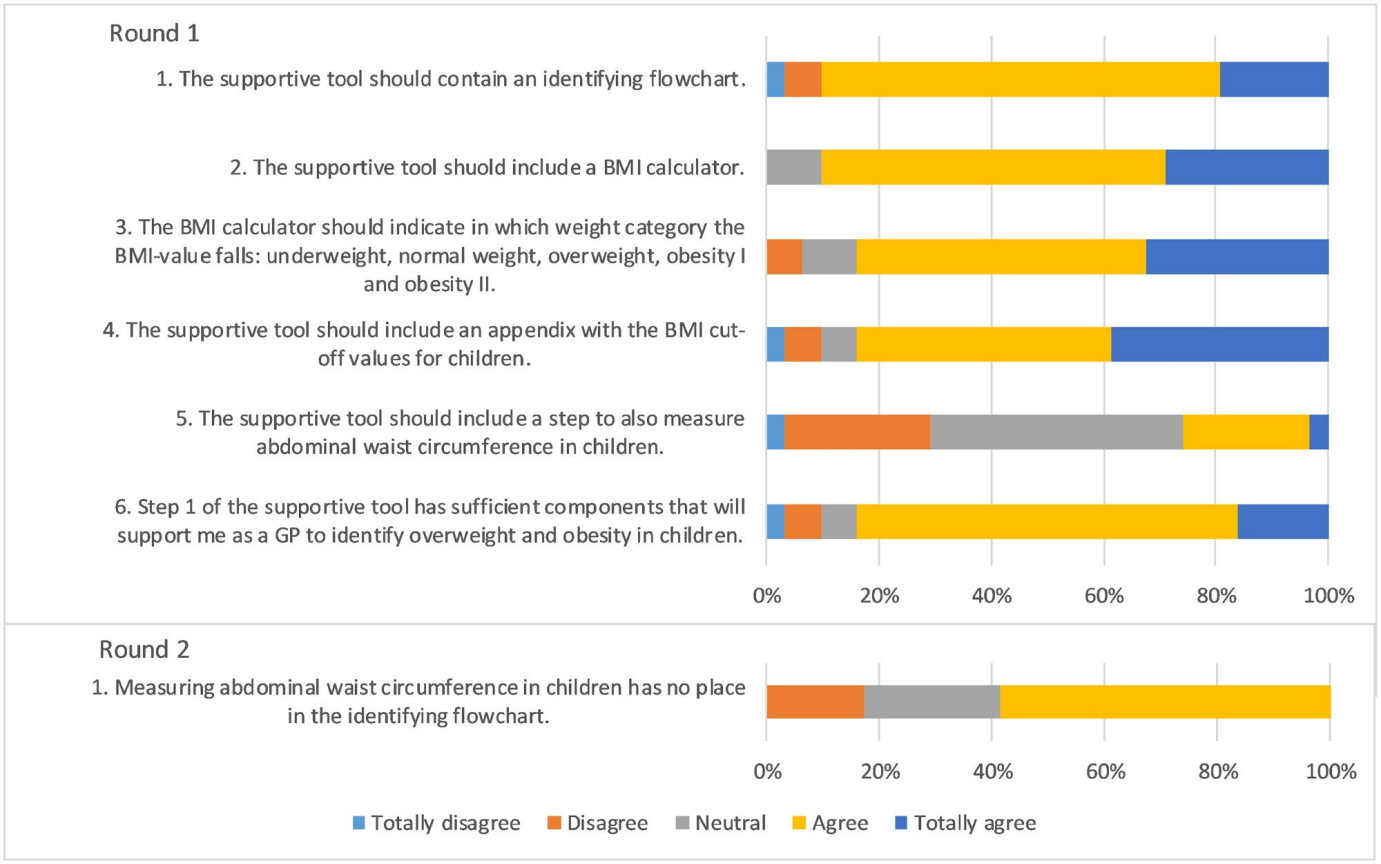
Outcomes statements on identifying overweight and obesity in children.

In the first round, consensus was reached for five out of six statements (Figure 1). Experts agreed that the tool should include a BMI calculator that clearly indicates whether the BMI value falls within the overweight or obesity category. During round two it was determined that waist circumference (WC) measurement in children should not be included in the tool.

### Statements about initiating

**Figure 2.**
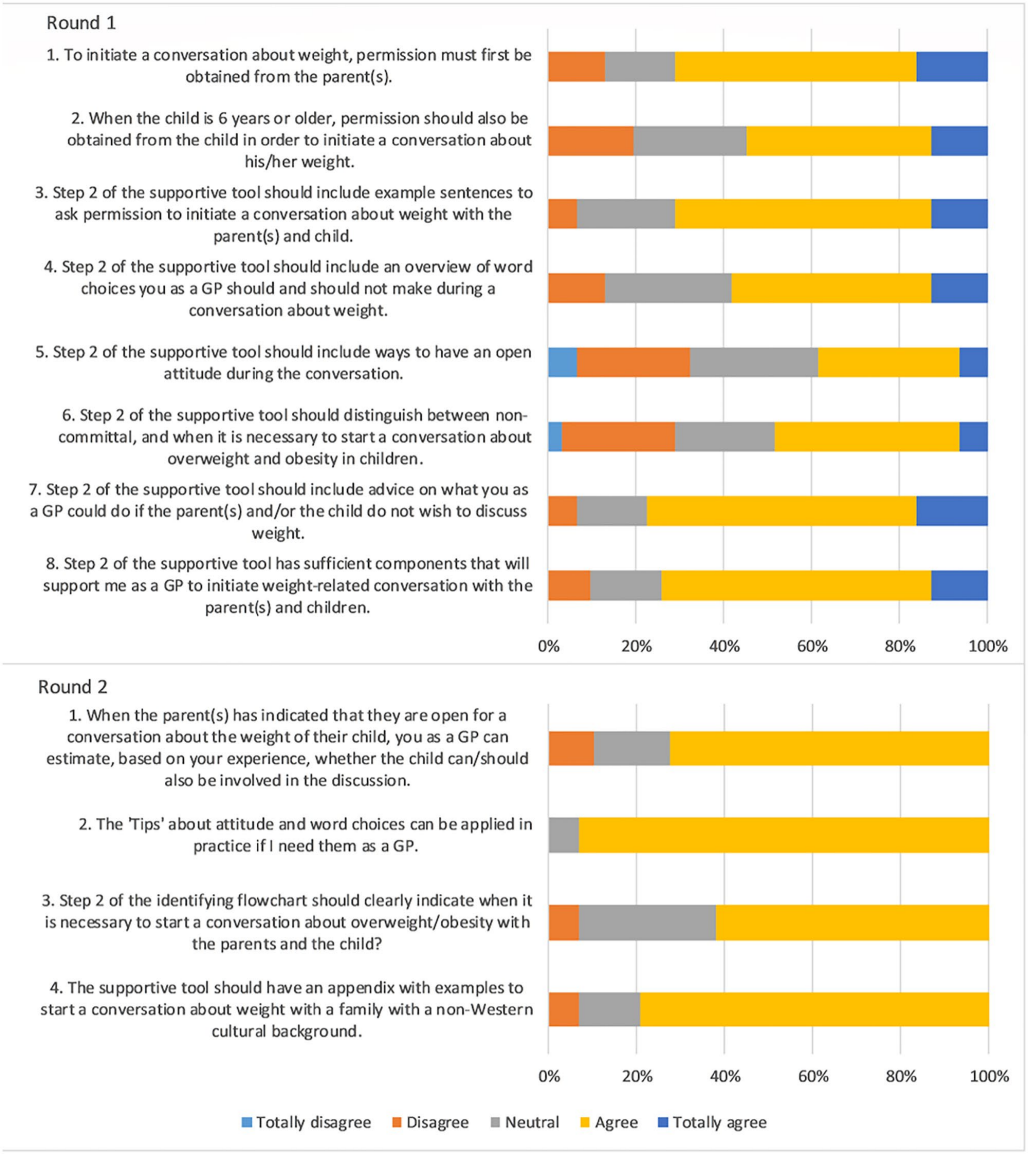
Outcomes statements on initiating weight-related conversations.

In round one consensus was reached for four of eight statements. Experts agreed that permission should be asked to parents and that the tool should include examples to initiate weight-related conversations. In round two consensus was reached for three of four modified statements (Figure 2). No consensus was reached for the statement if the tool should distinguish if it is necessary or not-committal to initiate a weight-related conversation with parents. Experts noted that it is always important to discuss overweight and obesity in children and that no distinction can be made whether it is necessary or not to mention.

### Statements about continuing

**Figure 3.**
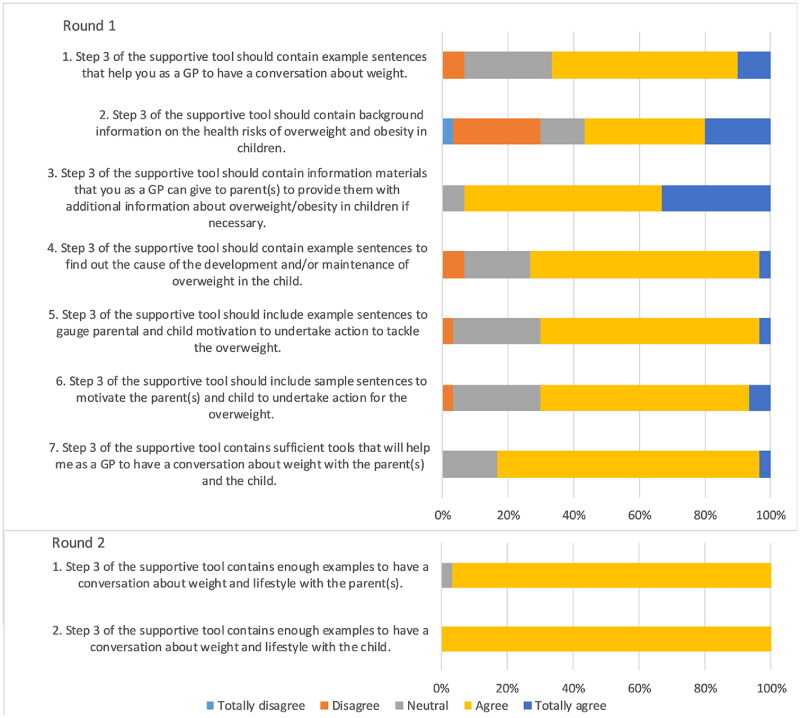
Outcomes statements on continuing weight-related conversations.

In two rounds, consensus was reached for all seven statements (Figure 3). Experts agreed that the tool should contain examples to continue weight-related conversations after initiating it with parents and children. The tool should include ways to figure out the cause and maintenance of overweight. Experts desire resources to inform families about health risks of overweight during childhood and the importance of a healthy lifestyle.

### Statements about referring

**Figure 4.**
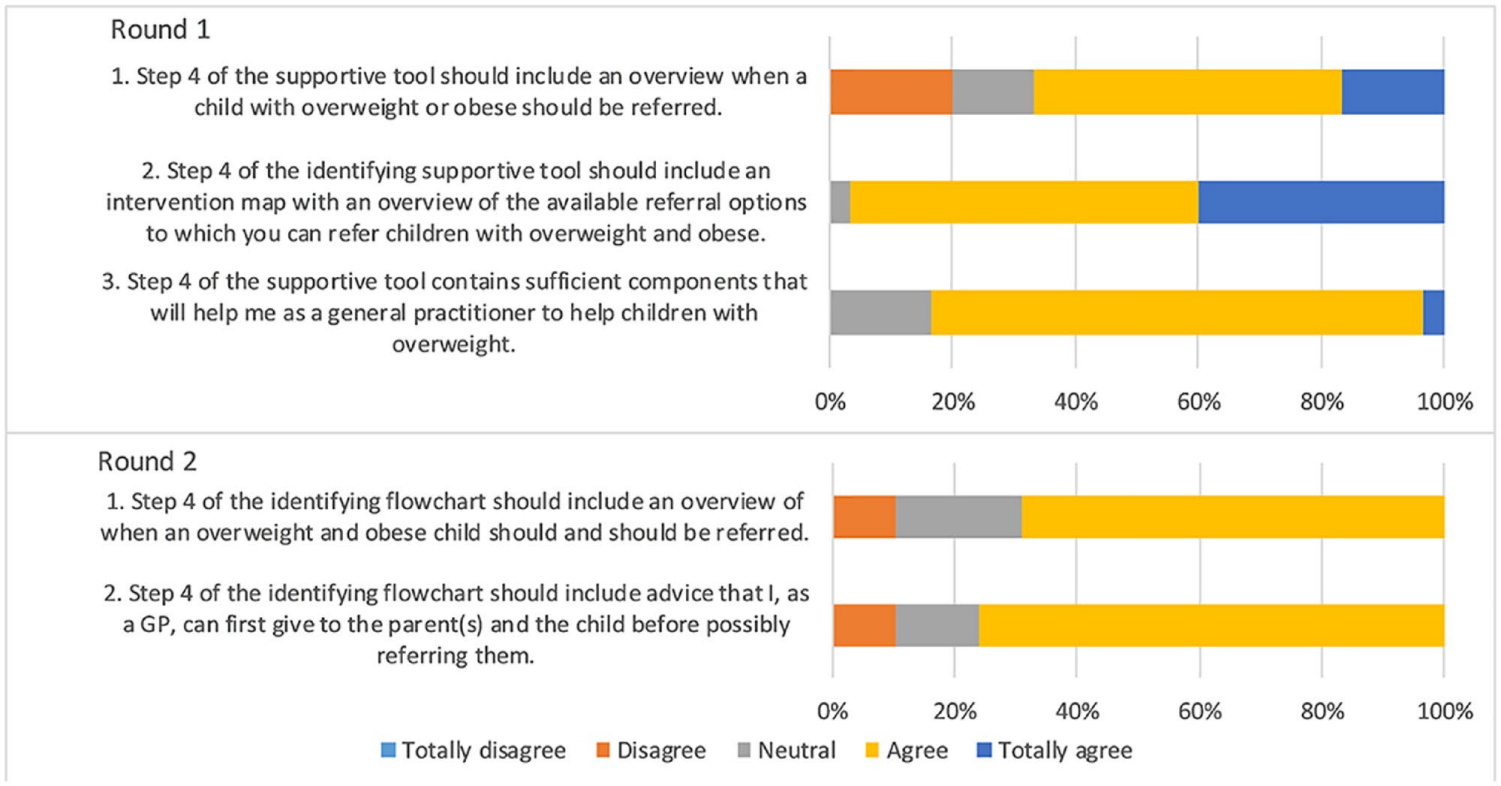
Outcomes statements on referring children with overweight and obesity.

Consensus was reached for the statements that the tool should contain an overview of available referral options for children with overweight and obesity (Figure 4). Experts noted that referrals should not be mandatory as healthy lifestyle advice can often be beneficial and therefore referral is not always necessary.

### Statements about the manual

**Figure 5.**
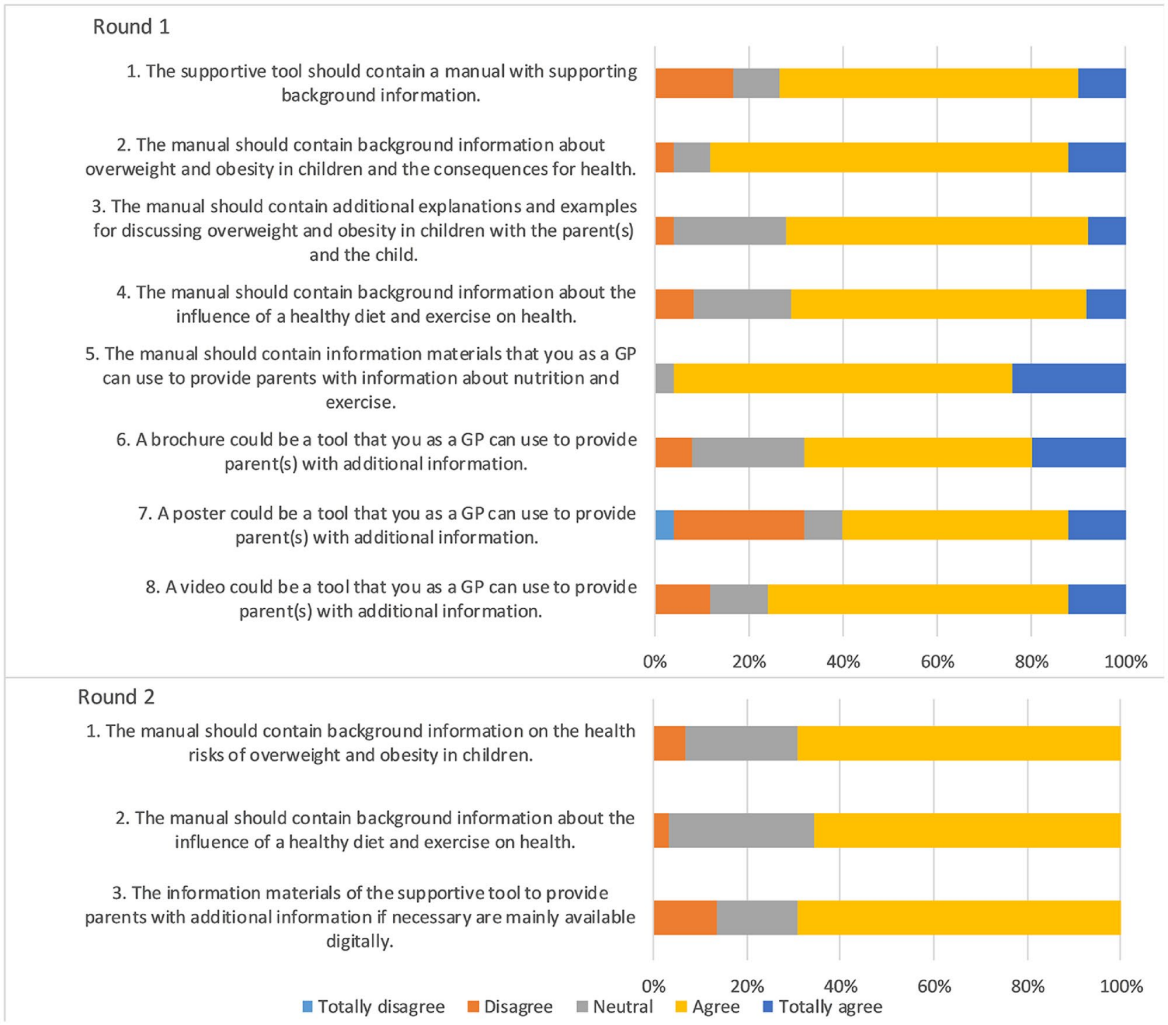
Outcomes statements on a tool manual.

Consensus was reached for five of seven statements in round one. In round two, no consensus was reached for three statements since the percentage ranged between 65.5% and 69.0% (Figure 5). The panel indicated that they had no specific preference for these topics. They noted that it would be acceptable if information about health risks and healthy lifestyles would be included as optional content, accessible both in printed form and digitally.

## Discussion

### Main findings

This study shows that GPs deem it necessary for a supportive tool aimed to identify, address and refer children with overweight and obesity in general practice to incorporate: a child-specific BMI calculator that categorises BMI into normal weight, overweight or obesity; examples to initiate and to continue weight-related conversations with parents and children; a map providing an overview of available referral options; a manual including information on health risks associated with overweight and obesity during childhood, healthy lifestyle advices and information materials.

### Strengths and limitations

A strength of this study was the use of the Delphi process as this is an appropriate methodology to achieve consensus by using multiple rounds of online surveys among experts within a short time frame in order to maintain engagement. This last resulted in a high response rate which is a strength as almost all experts completed both rounds. Whereas most Delphi studies perform three or more rounds, only two rounds were performed in this study. After two rounds, the agreement percentages for the six remaining statements ranged between 65.5–69.0%. These statements primarily concerned the necessary information for the tool manual. Most GPs who rated these statements as neutral, mentioned that the topics could be informative if they were optional to view. Based on participant’s suggestions, the research team decided to include these topics. Consequently, a third round was not conducted to avoid sample fatigue and reduce the burden on the experts. A limitation of this study is that no characteristics were collected from the participating GPs. Moreover, only GPs were included in this study, though perspectives of practice nurses and parents were included during earlier performed focus-group interviews and these outcomes were used for the development of the tool’s content [10].

### Comparison with existing literature

In round one, no consensus was reached regarding the statement that WC measurement should be used alongside BMI values to identify overweight and obesity in children. Although BMI is the most commonly used metric to identify overweight and obesity, it fails to show abdominal fat tissue which is an important risk factor for comorbidities [19]. Therefore, it was proposed that WC is a superior measurement to diagnose overweight and obesity in children, as it provides a better insight into abdominal fat and therefore into children’s health status [19–21]. However, GPs in present study mentioned that they found it challenging to perform WC measurement as it is unclear how to accurately conduct this in children. Furthermore, Dutch guidelines for GPs state that it remains unclear whether the additional step to perform WC measurement in children under 12 years old offers more value alongside the BMI value [6]. Interestingly, a feasibility study concerning an online tool [22] and a Delphi study on the development of a tool [23] for GPs in the management of overweight and obesity in children in the UK both showed that a child specialised BMI calculator was desired by GPs and clarified the severity of the overweight for parents. In line with GPs preferences and Dutch guidelines, it is recommended to not include WC measurement into a supportive tool for GPs intended to identify overweight in children aged 4–12 years. Though, GPs should be aware that not performing WC measurement could lead to miscategorisation of children with overweight as BMI does not differentiate between fat and muscle tissue.

Literature shows that cultural factors may be associated with the development of overweight and obesity in children [24]. Besides the high number of overweight and obesity in children of Dutch origin, there is an even higher prevalence of these conditions in children with a non-western background in the Netherlands [19]. In line with other studies, GPs in this study demand information and tools to manage overweight and obesity in children with a non-western background since differences in cultural background are perceived as main barriers to address overweight in children [1,7]. Moreover, differences in ethnic origin should be taken into account in the diagnosis of overweight and obesity, as specific growth charts exist for children of Turkish or Moroccan descent [6,25]. Hence, it is advised that supportive tools incorporate provisions for ethnical and cultural diversity. However, so far little is known about perceptions on overweight in various cultures [26]. A deeper understanding of these perceptions and weight norms is required to ensure the supportive tool is applicable to families from diverse cultural backgrounds.

In the Netherlands, the registered number of children with overweight and obesity referred to interventions by GPs is very low [27]. In line with multiple studies, this study shows that GPs need referral resources and desire an overview of appropriate interventions [13,14,22]. Interestingly, Sturgiss et al. (2017) showed that GPs felt more confident and were more likely to initiate weight-related conversations when equipped with a toolkit including examples to conduct weight-related discussions with patients. Therefore, we anticipate that providing GPs with a tool including a comprehensive map detailing available interventions and referral options will facilitate referrals and may increase referral rates of children with overweight and obesity in general practice settings [17,27].

No clear consensus was reached about statements whether the tool should contain information about health risks of childhood overweight and obesity and the importance of a healthy lifestyle. In line with literature, some GPs in this study indicated that this information is seen as basic knowledge [25]. However, several GPs pointed that medical training rarely covers healthy lifestyle advices or adverse health consequences of overweight and obesity in children. This aligns with recent publications showing that GPs often lack knowledge on these topics [16,28,29]. Therefore, a proportion of GPs desired inclusion of comprehensive information on lifestyle advices and health risks in the tool which aligns with literature [30]. Thus, information and recommendations on lifestyle and health risks of overweight during childhood should be included in the tool to meet the needs of most GPs.

### Implications for future research or clinical practice

This study provides insights in the required topics to develop a tool to support GPs to identify, address and refer children with overweight and obesity in general practice. The next step involves refining these topics by creating questions that both parents and children can easily understand, which will provide valuable insights for GPs on this issue, and designing an intervention map that outlines referral options. To further refine the tool, its feasibility when utilised by GPs in daily practice must be evaluated. Therefore, a pilot study is planned to investigate whether the tool indeed supports GPs and leads to the intended outcomes of more identifying, addressing and referral of children with overweight and obesity in practice. These findings are important to further optimise the tool and to implement it in general practice within the Netherlands and other countries with similar general practice settings.

## Conclusion

This Delphi study established consensus on the content topics to develop a supportive tool for GPs to identify, address and refer children with overweight and obesity in practice. Further research is needed to evaluate the feasibility of such a tool in daily general practice.

## Supplementary Material

Supplemental Material

Supplemental Material

## Data Availability

Data is available in this manuscript and the supplementary file. Any additional, sharable, data is available upon request from the authors.

## Social media handles (LinkedIn)

Maxime van der Velden: https://www.linkedin.com/in/maxime-van-der-velden-75133b163/
