# Supplementary material for: The development of a tool for GPs to manage overweight and obesity in children: A Delphi study
Source: Eur J Gen Pract. 2024 Oct 21;30(1):2413877. doi: 10.1080/13814788.2024.2413877 (PMC11494705; doi:10.1080/13814788.2024.2413877)
Supplement: Supplemental Material [file IGEN_A_2413877_SM4141.docx]

**Appendix 1**

**Appendix 1A: Survey round one (translated)**

**Survey 1. A Minimal Intervention Strategy for children with overweight and obesity.**

Dear GP,

Thank you for participating in our research.

The aim of this study is to reach consensus on the content of a Minimal Intervention Strategy (MIS) for children with overweight and obese that can be applied in general practice.

We have developed a MIS based on the NHG guidelines, literature and group interviews with general practitioners, practice assistants and parents of children with and without overweight. The MIS is intended to support general practitioners in identifying overweight and obesity in children, to discuss weight with the parent(s) and the child, and to provide the child with the right help.

Through this survey we ask you to assess the importance of the content of the MIS for relevance and applicability. The MIS consists of two parts: a signalling flow diagram and a manual.

All questions are multiple choice and can be answered with only one selection. There is room for each question to leave a comment to explain your answer choice or to suggest suggestions for the MIS. We would like you to ask to please make use of this and explain your answer, as this additional information is very valuable to us.

Completing the survey takes 20-30 minutes. Your participation in the study and your responses are strictly confidential to the research team and will not be shared with outside parties or other study participants.

We would be very pleased if you could complete the survey within one month. Once the survey has been completed by all participants, we will process the findings. You will then receive a second survey to assess the modified MIS. We will also process the findings of the second round.

If you have any questions or comments, please contact [author’s information]
E-mail: [author’s information]

Thank you again for participating in our study. We greatly appreciate your contribution to the development of the MIS, which will support general practitioners in the future in identifying, discussing and referring overweight and obesity in children.

Yours sincerely,

[author’s information]

Personal information:
Name:
E-mail address:

Part 1: The signalling flowchart.
The first part concerns the signalling flow diagram. The signalling flowchart consists of four steps:

1) identify overweight and obesity in children,
2) discussing obesity in children with the parents and the child,
3) having a conversation about weight,
4) further helping the child and the parents.

The first part of the survey consists of four sub-parts in which you answer questions about each step of the signalling flow diagram. Below is the signalling flow diagram:


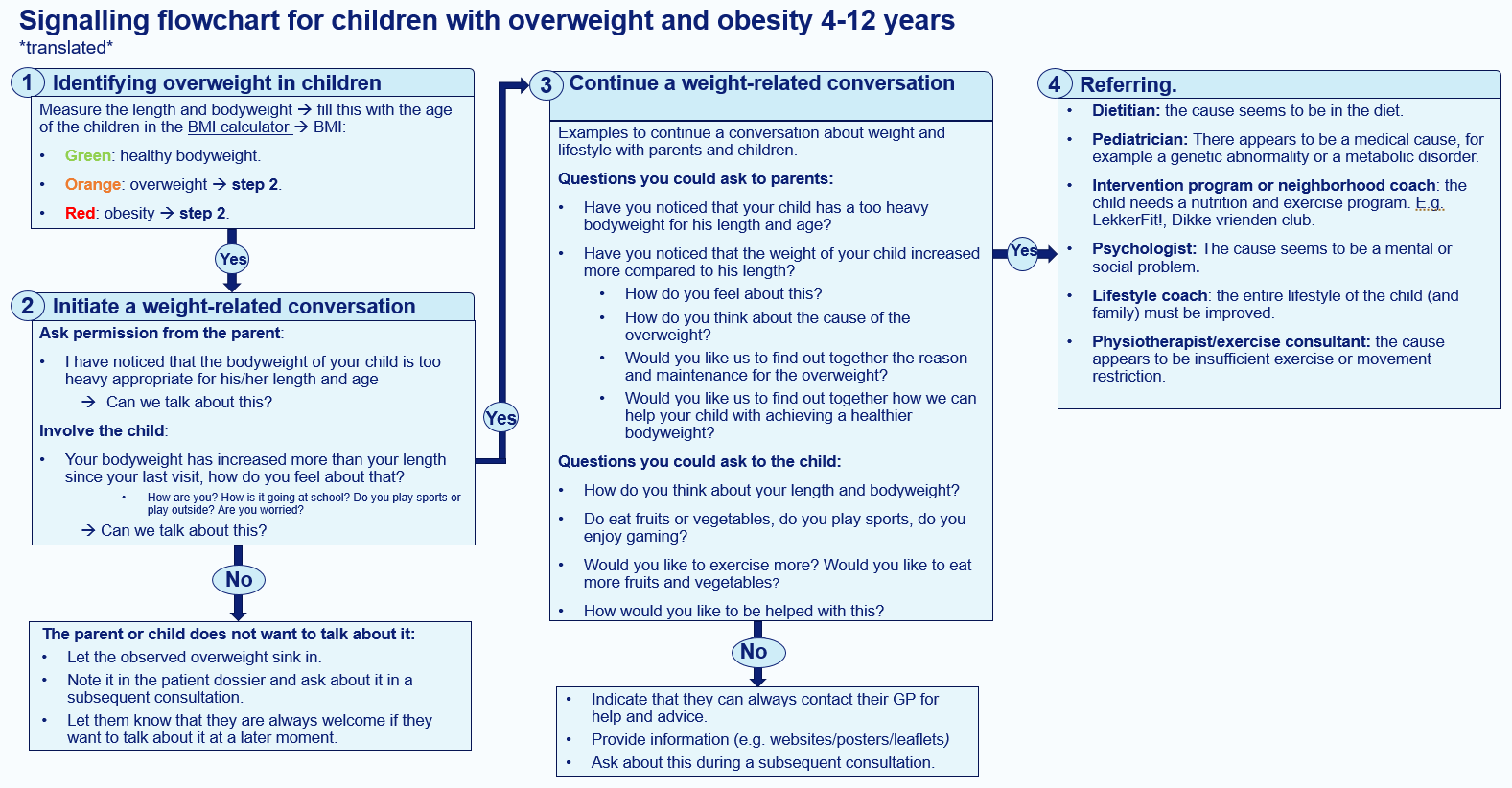


Subpart 1/4: Step 1. Has the child overweight?
The first subsection concerns Step 1 of the signalling flow diagram: Identifying overweight and obesity in children. This part consists of 6 questions. Please explain your answer choice.


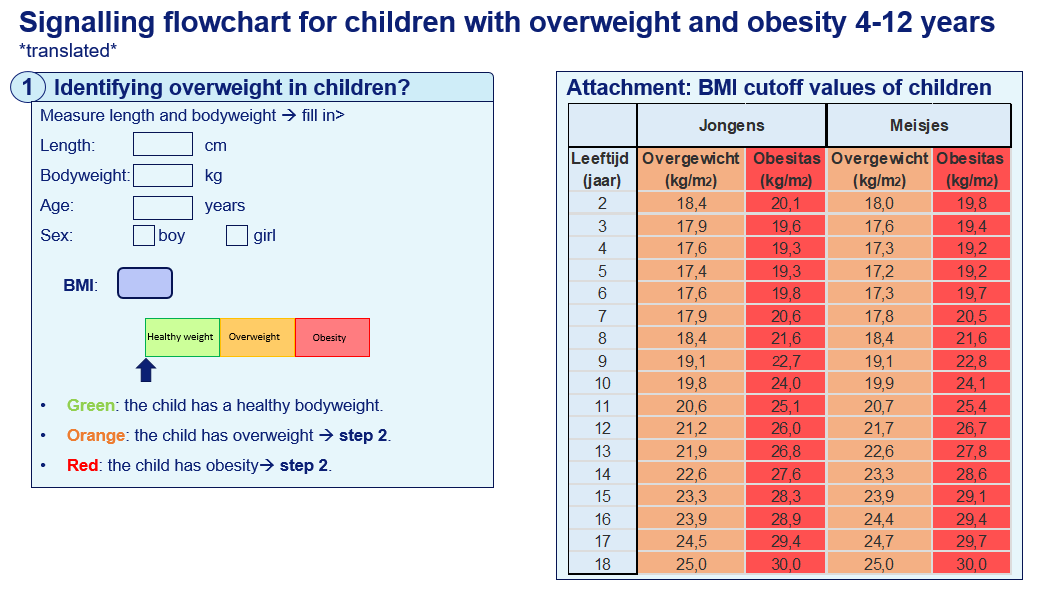


1. The supportive tool should contain a signalling flowchart.

| Totally disagree | Disagree | Neutral | Agree | Totally agree | |
| --- | --- | --- | --- | --- | --- |
|  |  |  |  |  | |
| Enter your comments here: | | | | |  |

2. The supportive tool should include a BMI calculator.

| Totally disagree | Disagree | Neutral | Agree | Totally agree | |
| --- | --- | --- | --- | --- | --- |
|  |  |  |  |  | |
| Enter your comments here: | | | | |  |

3. The BMI calculator should indicate in which weight category the BMI-value falls: underweight, normal weight, overweight, obesity I and obesity II.

| Totally disagree | Disagree | Neutral | Agree | Totally agree | |
| --- | --- | --- | --- | --- | --- |
|  |  |  |  |  | |
| Enter your comments here: | | | | |  |

4. The supportive tool should include an appendix with the BMI cut-off values for children.
‘i’ The BMI gives a good indication of whether someone has a healthy weight. Separate cut-off values apply for children per age and gender. The cut-off value is the chosen value up to which the weight falls into the category of underweight, normal weight, overweight or obese. The BMI calculator for children takes this into account.

| Totally disagree | Disagree | Neutral | Agree | Totally agree | |
| --- | --- | --- | --- | --- | --- |
|  |  |  |  |  | |
| Enter your comments here: | | | | |  |

5. The supportive tool should include a step to also measure abdominal waist circumference in children.

| Totally disagree | Disagree | Neutral | Agree | Totally agree | |
| --- | --- | --- | --- | --- | --- |
|  |  |  |  |  | |
| Enter your comments here: | | | | |  |

6. Step 1 of the supportive tool has sufficient components that will support me as a GP to signal overweight and obesity in children.
‘i’ This question is intended as a summary question. So step 1 contains sufficient tools that will help you as a GP to identify overweight and obesity in children, or are there still parts that you are missing.

| Totally disagree | Disagree | Neutral | Agree | Totally agree | |
| --- | --- | --- | --- | --- | --- |
|  |  |  |  |  | |
| Enter your comments here: | | | | |  |

Subpart 2/4: Step 2. Discussing obesity.
The second subsection concerns Step 2 of the signalling flow diagram: Discussing obesity with the parent(s) and the child. This part consists of 8 questions. Please explain your answer choice.


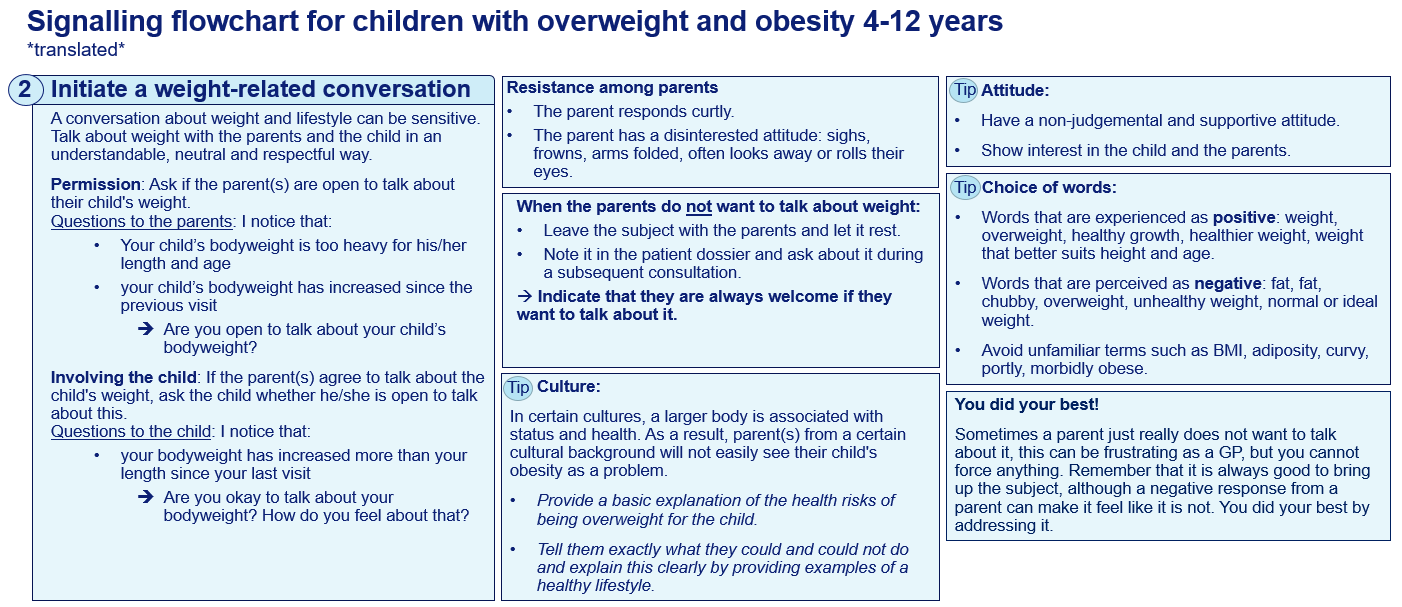


1. To initiate a conversation about weight, permission must first be obtained from the parent(s).

| Totally disagree | Disagree | Neutral | Agree | Totally agree | |
| --- | --- | --- | --- | --- | --- |
|  |  |  |  |  | |
| Enter your comments here: | | | | |  |

2. When the child is 6 years or older, permission should also be obtained from the child in order to initiate a conversation about his/her weight.

| Totally disagree | Disagree | Neutral | Agree | Totally agree | |
| --- | --- | --- | --- | --- | --- |
|  |  |  |  |  | |
| Enter your comments here: | | | | |  |

3. Step 2 of the supportive tool should include example sentences to ask permission to initiate a conversation about weight with the parent(s) and child.

| Totally disagree | Disagree | Neutral | Agree | Totally agree | |
| --- | --- | --- | --- | --- | --- |
|  |  |  |  |  | |
| Enter your comments here: | | | | |  |

4. Step 2 of the supportive tool should include an overview of word choices you as a GP should and should not make during a conversation about weight.

| Totally disagree | Disagree | Neutral | Agree | Totally agree | |
| --- | --- | --- | --- | --- | --- |
|  |  |  |  |  | |
| Enter your comments here: | | | | |  |

5. Step 2 of the supportive tool should include ways to have an open attitude during the conversation.

| Totally disagree | Disagree | Neutral | Agree | Totally agree | |
| --- | --- | --- | --- | --- | --- |
|  |  |  |  |  | |
| Enter your comments here: | | | | |  |

6. Step 2 of the supportive tool should distinguish between non-committal, and when it is necessary to start a conversation about overweight and obesity in children.

| Totally disagree | Disagree | Neutral | Agree | Totally agree | |
| --- | --- | --- | --- | --- | --- |
|  |  |  |  |  | |
| Enter your comments here: | | | | |  |

7. Step 2 of the supportive tool should include advice on what you as a GP could do if the parent(s) and/or the child do not wish to discuss weight.

| Totally disagree | Disagree | Neutral | Agree | Totally agree | |
| --- | --- | --- | --- | --- | --- |
|  |  |  |  |  | |
| Enter your comments here: | | | | |  |

8. Step 2 of the supportive tool has sufficient components that will support me as a GP to initiate weight-related conversation with the parent(s) and children.
‘i‘ This question is intended as a summary question. So step 2 contains sufficient tools that will help you as a GP to start a conversation about obesity with the parents and the child, or are there still parts that you are missing.

| Totally disagree | Disagree | Neutral | Agree | Totally agree | |
| --- | --- | --- | --- | --- | --- |
|  |  |  |  |  | |
| Enter your comments here: | | | | |  |

Subpart 3/4: Step 3. Have a conversation about overweight and obesity.
The third subsection concerns Step 3 of the signalling flow diagram: Having a conversation about weight with the parent(s) and the child. This part consists of 7 questions. Please explain your answer choice.


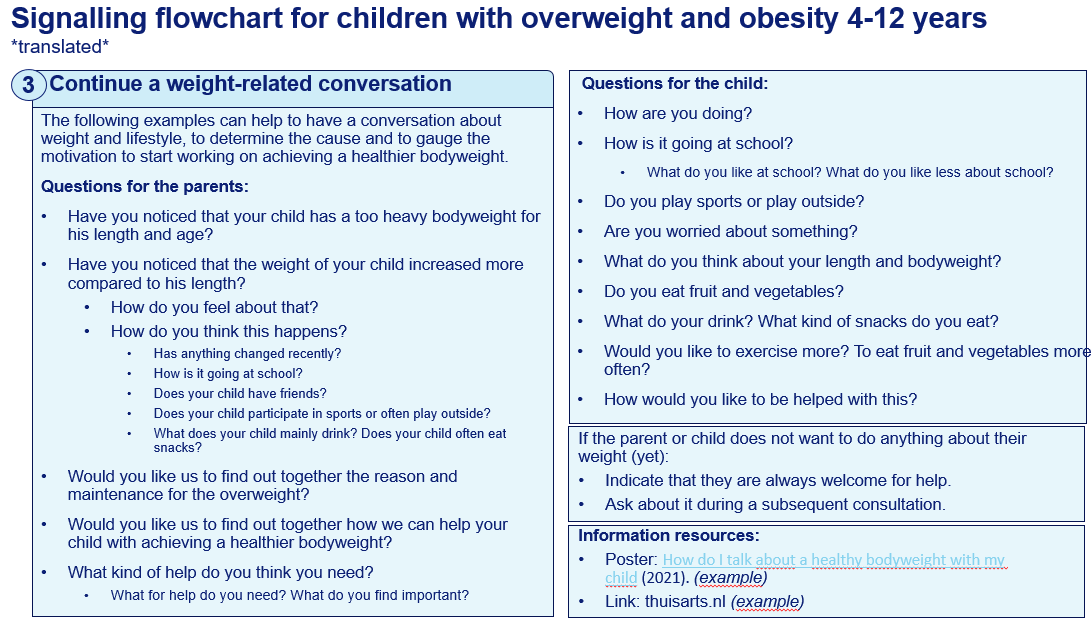


1. Step 3 of the supportive tool should contain example sentences that help you as a GP to have a conversation about weight.

| Totally disagree | Disagree | Neutral | Agree | Totally agree | |
| --- | --- | --- | --- | --- | --- |
|  |  |  |  |  | |
| Enter your comments here: | | | | |  |

2. Step 3 of the supportive tool should contain background information on the health risks of overweight and obesity in children.

| Totally disagree | Disagree | Neutral | Agree | Totally agree | |
| --- | --- | --- | --- | --- | --- |
|  |  |  |  |  | |
| Enter your comments here: | | | | |  |

3. Step 3 of the supportive tool should contain information materials that you as a GP can give to parent(s) to provide them with additional information about overweight/obesity in children if necessary.

| Totally disagree | Disagree | Neutral | Agree | Totally agree | |
| --- | --- | --- | --- | --- | --- |
|  |  |  |  |  | |
| Enter your comments here: | | | | |  |

4. Step 3 of the supportive tool should contain example sentences to find out the cause of the development and/or maintenance of overweight in the child.

| Totally disagree | Disagree | Neutral | Agree | Totally agree | |
| --- | --- | --- | --- | --- | --- |
|  |  |  |  |  | |
| Enter your comments here: | | | | |  |

5. Step 3 of the supportive tool should include example sentences to gauge parental and child motivation to undertake action to tackle the overweight.

| Totally disagree | Disagree | Neutral | Agree | Totally agree | |
| --- | --- | --- | --- | --- | --- |
|  |  |  |  |  | |
| Enter your comments here: | | | | |  |

6. Step 3 of the supportive tool should include sample sentences to motivate the parent(s) and child to undertake action for the overweight.

| Totally disagree | Disagree | Neutral | Agree | Totally agree | |
| --- | --- | --- | --- | --- | --- |
|  |  |  |  |  | |
| Enter your comments here: | | | | |  |

7. Step 3 of the supportive tool contains sufficient tools that will help me as a GP to have a conversation about weight with the parent(s) and the child.
‘i‘ This question is intended as a summary question. So step 3 contains sufficient tools that will help you as a GP to have a conversation about obesity with the parents and the child, or are there still parts that you are missing.

| Totally disagree | Disagree | Neutral | Agree | Totally agree | |
| --- | --- | --- | --- | --- | --- |
|  |  |  |  |  | |
| Enter your comments here: | | | | |  |

Subpart 4/4: Step 4. Refer.
The fourth subsection concerns step 4 of the signalling flowchart: Referral. This part consists of 3 questions. Please explain your answer choice.


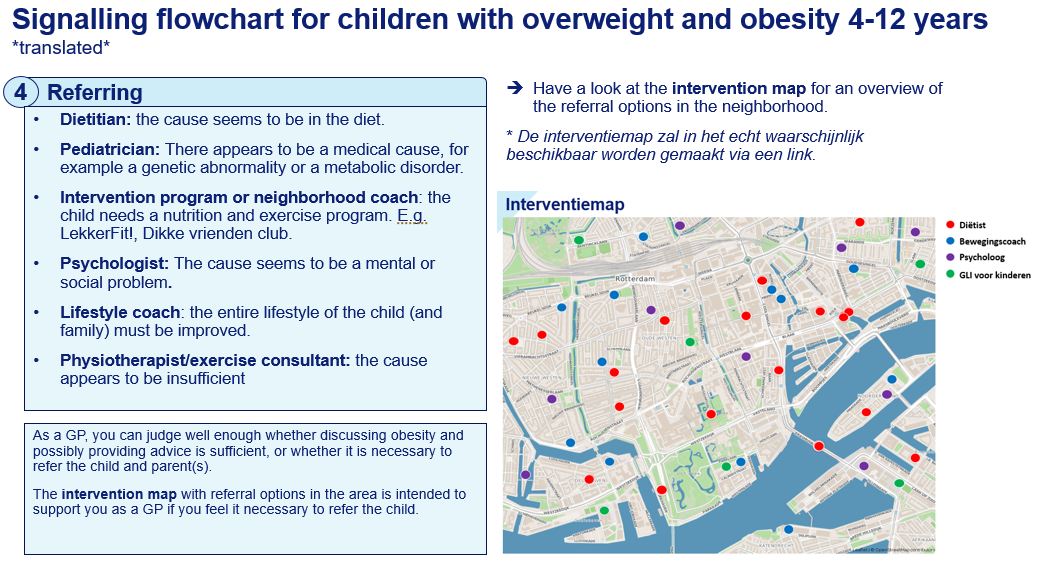


1. Step 4 of the supportive tool should include an overview when a child with overweight or obese should be referred.

| Totally disagree | Disagree | Neutral | Agree | Totally agree | |
| --- | --- | --- | --- | --- | --- |
|  |  |  |  |  | |
| Enter your comments here: | | | | |  |

2. Step 4 of the signalling supportive tool should include an intervention map with an overview of the available referral options to which you can refer children with overweight and obese.
‘i’ The intervention map is an online overview of the available referral options for overweight and obese children at district level in the Rotterdam region.

| Totally disagree | Disagree | Neutral | Agree | Totally agree | |
| --- | --- | --- | --- | --- | --- |
|  |  |  |  |  | |
| Enter your comments here: | | | | |  |

3. Step 4 of the supportive tool contains sufficient components that will help me as a general practitioner to help children with overweight.
‘i’ This question is intended as a summary question. So step 4 contains sufficient tools that will help you as a GP to refer children with overweight and obesity.

| Totally disagree | Disagree | Neutral | Agree | Totally agree | |
| --- | --- | --- | --- | --- | --- |
|  |  |  |  |  | |
| Enter your comments here: | | | | |  |

Part 2: The MIS manual The second part concerns the MIS Manual.
The manual will contain background information and serves as a reference. This part consists of 8 questions.
! If your answer choice for question 1 is neutral, agree, or strongly agree, please answer questions 2 through 8.
! If your answer choice for question 1 is strongly disagree or disagree, you do not have to answer questions 2 to 8 and you can click send.

1. The supportive tool should contain a manual with supporting background information.

| Totally disagree | Disagree | Neutral | Agree | Totally agree | |
| --- | --- | --- | --- | --- | --- |
|  |  |  |  |  | |
| Enter your comments here: | | | | |  |

2. The manual should contain background information about overweight and obesity in children and the consequences for health.

| Totally disagree | Disagree | Neutral | Agree | Totally agree | |
| --- | --- | --- | --- | --- | --- |
|  |  |  |  |  | |
| Enter your comments here: | | | | |  |

3. The manual should contain additional explanations and examples for discussing overweight and obesity in children with the parent(s) and the child.

| Totally disagree | Disagree | Neutral | Agree | Totally agree | |
| --- | --- | --- | --- | --- | --- |
|  |  |  |  |  | |
| Enter your comments here: | | | | |  |

4. The manual should contain background information about the influence of a healthy diet and exercise on health.

| Totally disagree | Disagree | Neutral | Agree | Totally agree | |
| --- | --- | --- | --- | --- | --- |
|  |  |  |  |  | |
| Enter your comments here: | | | | |  |

5. The manual should contain information materials that you as a GP can use to provide parents with information about nutrition and exercise.

| Totally disagree | Disagree | Neutral | Agree | Totally agree | |
| --- | --- | --- | --- | --- | --- |
|  |  |  |  |  | |
| Enter your comments here: | | | | |  |

6. A brochure could be a tool that you as a GP can use to provide parent(s) with additional information.

| Totally disagree | Disagree | Neutral | Agree | Totally agree | |
| --- | --- | --- | --- | --- | --- |
|  |  |  |  |  | |
| Enter your comments here: | | | | |  |

7. A poster could be a tool that you as a GP can use to provide parent(s) with additional information.

| Totally disagree | Disagree | Neutral | Agree | Totally agree | |
| --- | --- | --- | --- | --- | --- |
|  |  |  |  |  | |
| Enter your comments here: | | | | |  |

8. A video could be a tool that you as a GP can use to provide parent(s) with additional information.

| Totally disagree | Disagree | Neutral | Agree | Totally agree | |
| --- | --- | --- | --- | --- | --- |
|  |  |  |  |  | |
| Enter your comments here: | | | | |  |

Thank you for completing the questionnaire.

We will process all answers. You will hear from us again when another survey is required.

Yours sincerely,

[author’s information]

**Appendix 1B: Survey round one (translated)**

**Survey 2. A Minimal Intervention Strategy for children with overweight and obesity.**

Dear GP,

First of all, thank you very much for participating and completing the first questionnaire.

The input from the first survey helped us a lot in adjusting the MIS. Consensus has already been reached on many topics and it has become clear that they can be included in the MIS, whether or not in an adapted form, or are unnecessary.

No consensus has yet been reached on a number of topics. We have adjusted these topics based on all the comments and would like to present them to you again.

The email you received from us with the link to the second survey also contains an attachment with the results of the first survey. In this document you can see how opinions were divided in the first round.

All questions are multiple choice questions and can be answered with only one selection. There is room for each question to leave a comment to explain your answer choice or to suggest suggestions. We would like to expressly ask you to make use of this. This survey is shorter than the first questionnaire. Completing the form takes approximately 10-15 minutes.

Your participation in the study and your responses are strictly confidential to the research team and will not be shared with outside parties or other study participants.

If you have any questions or comments, please contact: [author’s information]

E-mail: [author’s information]

Thank you again for participating in our research.

Yours sincerely,

[author’s information]

Personal information:
Name:
E-mail address:

Part 1: The signalling flowchart.
The first part concerns the signalling flow diagram. The signalling flowchart consists of four steps:

1) identify overweight and obesity in children,
2) discussing obesity in children with the parents and the child,
3) having a conversation about weight,
4) further helping the child and the parents.

The first part of the questionnaire consists of four sub-parts in which you answer questions about each step of the signalling flow diagram. Below is the signalling flow diagram:


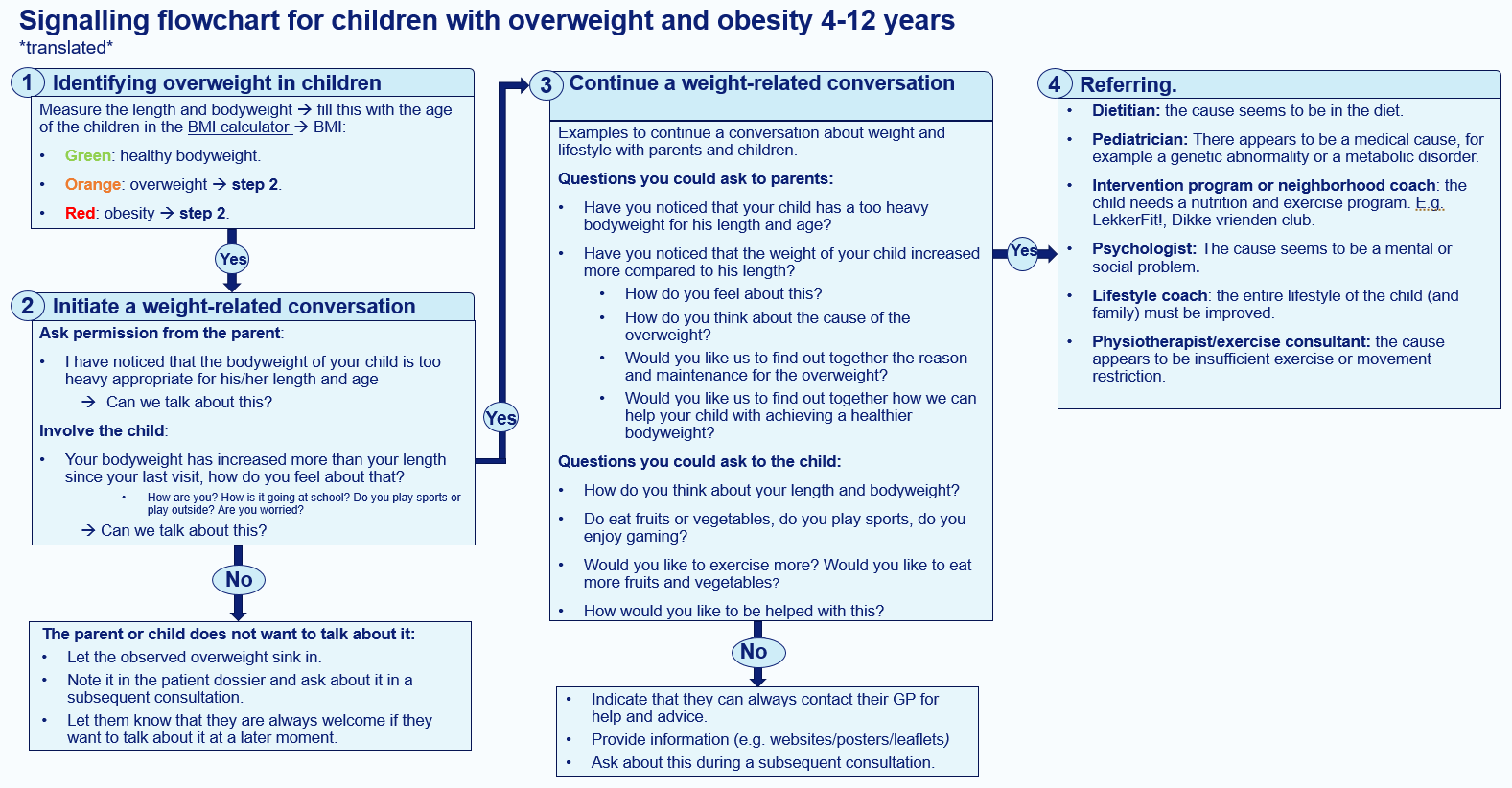


Subpart 1/4: Step 1. Is the child overweight?
The first subsection concerns Step 1 of the signalling flow diagram: Identifying overweight and obesity in children. This part consists of 1 question. Please explain your answer choice

1. Measuring abdominal waist circumference in children has no place in the signalling flowchart.

| Totally disagree | Disagree | Neutral | Agree | Totally agree | |
| --- | --- | --- | --- | --- | --- |
|  |  |  |  |  | |
| Enter your comments here: | | | | |  |

Subpart 2/4: Step 2. Discussing obesity.
The second subsection concerns Step 2 of the signaling flow diagram: Discussing obesity with the parent(s) and the child. This part consists of 4 questions. Please explain your answer choice.

1. When the parent(s) has indicated that they are open for a conversation about the weight of their child, you as a GP can estimate, based on your experience, whether the child can/should also be involved in the discussion.

| Totally disagree | Disagree | Neutral | Agree | Totally agree | |
| --- | --- | --- | --- | --- | --- |
|  |  |  |  |  | |
| Enter your comments here: | | | | |  |

2. The 'Tips' about attitude and word choices can be applied in practice if I need them as a GP.

| Totally disagree | Disagree | Neutral | Agree | Totally agree | |
| --- | --- | --- | --- | --- | --- |
|  |  |  |  |  | |
| Enter your comments here: | | | | |  |

3. Step 2 of the signalling flowchart should clearly indicate when it is necessary to start a conversation about overweight/obesity with the parents and the child?

| Totally disagree | Disagree | Neutral | Agree | Totally agree | |
| --- | --- | --- | --- | --- | --- |
|  |  |  |  |  | |
| Enter your comments here: | | | | |  |

4. The supportive tool should have an appendix with examples to start a conversation about weight with a family with a non-Western cultural background.

| Totally disagree | Disagree | Neutral | Agree | Totally agree | |
| --- | --- | --- | --- | --- | --- |
|  |  |  |  |  | |
| Enter your comments here: | | | | |  |

Subpart 3/4: Step 3. Have a conversation about overweight and obesity.
The third subsection concerns Step 3 of the signaling flow diagram: Having a conversation about weight with the parent(s) and the child. This part consists of 2 questions. Please explain your answer choice.

1. Step 3 of the supportive tool contains enough examples to have a conversation about weight and lifestyle with the parent(s).

| Totally disagree | Disagree | Neutral | Agree | Totally agree | |
| --- | --- | --- | --- | --- | --- |
|  |  |  |  |  | |
| Enter your comments here: | | | | |  |

2. Step 3 of the supportive tool contains enough examples to have a conversation about weight and lifestyle with the child.

| Totally disagree | Disagree | Neutral | Agree | Totally agree | |
| --- | --- | --- | --- | --- | --- |
|  |  |  |  |  | |
| Enter your comments here: | | | | |  |

Subpart 4/4: Step 4. Refer.
The fourth subsection concerns step 4 of the signalling flowchart: Referral. This part consists of 2 questions. Please explain your answer choice.

1. Step 4 of the signalling flowchart should include an overview of when an overweight and obese child should and should be referred.

| Totally disagree | Disagree | Neutral | Agree | Totally agree | |
| --- | --- | --- | --- | --- | --- |
|  |  |  |  |  | |
| Enter your comments here: | | | | |  |

2. Step 4 of the signalling flowchart should include advice that I, as a GP, can first give to the parent(s) and the child before possibly referring them.

| Totally disagree | Disagree | Neutral | Agree | Totally agree | |
| --- | --- | --- | --- | --- | --- |
|  |  |  |  |  | |
| Enter your comments here: | | | | |  |

Part 2: The MIS manual
The second part concerns the MIS Manual. The manual will contain background information and serves as a reference. This part consists of 3 questions. Please explain your answer choice.

1. The manual should contain background information on the health risks of overweight and obesity in children.

| Totally disagree | Disagree | Neutral | Agree | Totally agree | |
| --- | --- | --- | --- | --- | --- |
|  |  |  |  |  | |
| Enter your comments here: | | | | |  |

2. The manual should contain background information about the influence of a healthy diet and exercise on health.

| Totally disagree | Disagree | Neutral | Agree | Totally agree | |
| --- | --- | --- | --- | --- | --- |
|  |  |  |  |  | |
| Enter your comments here: | | | | |  |

3. The information materials of the supportive tool to provide parents with additional information if necessary are mainly available digitally.

| Totally disagree | Disagree | Neutral | Agree | Totally agree | |
| --- | --- | --- | --- | --- | --- |
|  |  |  |  |  | |
| Enter your comments here: | | | | |  |
